# Economically Feasible Wood Biopreservation Platform in *Lannea coromandelica* (Houtt.) Merr. Against Wood Rotting Fungus Through Bio-Prospecting Weed Extracts

**DOI:** 10.3389/fpls.2021.696747

**Published:** 2021-07-16

**Authors:** Heena Gupta, Kulwant Rai Sharma, J. N. Sharma

**Affiliations:** ^1^Department of Forest Products, College of Forestry, Dr. Y. S. Parmar University of Horticulture and Forestry Nauni, Solan, India; ^2^Department of Plant Pathology, College of Horticulture, Dr. Y. S. Parmar University of Horticulture and Forestry Nauni, Solan, India

**Keywords:** *Lannea coromandelica*, antifungal, dimensional stability, durability, preservative, wood

## Abstract

As an alternative to synthetic preservatives, the use of plant-based, environmentally sustainable preservatives for wood protection has tremendous potential. The current research analyzed the dimensional stability and longevity of *Lannea coromandelica* wood using weed extracts viz. *Lantana camara* L. and *Ageratum conyzoides* L., respectively. Petroleum ether (PE) and methanolic weed extracts were used to treat wood blocks (5 cm × 2.5 cm × 2.5 cm) at varying concentrations ranging from 0.25 to 2.00%. The PE extract of *A. conyzoides* resulted in maximum swelling (tangential plane, 6.30%) at 2.00%, volumetric swelling coefficient (13.17%) at 1.50%, and volumetric shrinkage coefficient (7.71%) at 1.00% concentration, while maximum shrinkage (tangential plane, 4.10%) in methanol (M) extract was observed. In *L. camara* methanolic extract (1.00%), maximum anti-shrink efficiency (37.01%) was recorded. *In vitro* mycelial growth of the wood-rotting fungus was completely inhibited by PE extract from both weeds. However, the methanolic extract of *A. conyzoides* resulted in maximal inhibition (75.93%) at a concentration of 2.00%. Also, PE extract (2.00%) of *A. conyzoides* reduced the fungal colonization to 50%, as compared with control. The lowest weight loss (decay test, 12 weeks) was observed at a 2.00% concentration of *L. camara* PE extract. The present research highlighted that both *A. conyzoides* and *L. camara* could be used as an environmentally sustainable wood preservative substitute that will encourage the utilization of *L. coromandelica* in wood-based industries.

## Introduction

Wood is one of the most important and versatile natural resources of humanity, assisting various nations in achieving sustainable development and improving technology and welfare (Daly-Hassen et al., 2014; Verhaegen et al., 2014). The strong mechanical properties of wood and its ease of processing and satisfying aesthetic appearance have not only led to its widespread use in tools, furniture, buildings, and decorations (Qiu et al., 2018) but also in the emerging fields of transparent materials (Zhu et al., 2016, Yu et al., 2017), water clean-up and extraction (Liu et al., 2017; Zhu et al., 2017; Wang et al., 2019), energy storage (Yang et al., 2018), stimuli-responsive materials (Li et al., 2018), and electronic devices (Chen et al., 2018). Since wood, underexposed environmental conditions, is vulnerable to deterioration caused by various organisms and abiotic influences, it must have high natural durability for these applications. The constitutive biopolymers (cellulose, lignin, and hemicelluloses) of wood are subjected to intense and progressive oxidative degradation processes (photo-oxidation, chemical oxidation, thermal decomposition, and photolysis reactions) as a result of environmental factors (sunlight radiation, primarily UV component; moisture produced by dewing, raining, and snowing; chemical pollutants; fire; heat/cold variations; wind abrasion-particulates; atmospheric oxygen), which affects the natural durability of the wood (dimensional stability, surface integrity) and causes significant structural and color changes (discoloration), as well as a progressive reduction in its resistance to biological agents (biodegradation or decay development) and mechanical properties (Teaca et al., 2019).

Several wood species (*Tectona grandis, Cedrus deodara*, etc.) have very good natural resilience against destructive wood agents, but global demand has outstripped supply or availability in the marketplace. Furthermore, timber production from government forest areas in India accounts for 3.35% of total demand, or 153 million m^3^ in 2020 (projected), while potential timber production accounts for 45% of total demand for raw wood by various wood-based industries (Brocco et al., 2017; Vanam, 2019). The decline in raw material supply, especially for traditional/primary timber species, is continually impeding the production activities of wood-based industries, resulting in limited output and growth globally (Purnomo et al., 2011; Zhou et al., 2015; Antwi-Boasiako and Boadu, 2016). Importing raw materials could solve the problem, but it would raise the cost of operations and goods, slowing the development of local industries. To ensure a consistent supply of raw materials, one of the best strategies would be introducing lesser used timber species (LUS) into the market, as LUS is abundant in many sustainably managed tropical forests, lowering costs (Antwi-Boasiako and Boadu, 2016).

As a result, *Lannea coromandelica*, a fast-growing deciduous tropical tree in the Anacardiaceae family widely distributed in waste places and forests throughout India, Bangladesh, and some other tropical countries, was chosen for this reason (Reddy et al., 2011; Weerapreeyakul et al., 2016). However, despite having a density of 0.77 gm/cm^3^ (at 12% MC), this species is rated as non-durable or non-resistant to natural decay agents as resistance to natural decay depends on higher extractive content rather than the higher density.

Furthermore, the difficulty in seasoning has limited the use of the species in furniture production, house building, and other structural purposes (Rahman et al., 2013). However, synthetic wood preservatives can enhance its durability for a variety of end-uses. While chemical preservation of wood is one of the most effective methods for inducing dimensional stability, UV resistance, and biological resistance in wood (Rowell, 2005), it is costly and becoming increasingly restricted due to both pronounced toxicity and harmful environmental effects (Kartal et al., 2015; Teaca et al., 2019). Furthermore, the treated wood needs maintenance throughout its existence, posing a risk in the disposal and reuse of this material (Wang et al., 2016). Concerns about the environmental effects of conventional wood preservatives have fueled the need for the production of alternative wood protection agents and methods based on natural materials that are both cost-effective and environmentally friendly (Edlich et al., 2005; Singh and Singh, 2012; Mohammed et al., 2016; Tchinda et al., 2018). The high availability and rapid proliferation of invasive plants (e.g., *Ageratum conyzoides, Eupatorium* sp., *Lantana camara, Mikania micrantha*, and *Parthenium hysteophorus*) will not only improve the overall economic feasibility of the process but may also solve the problems associated with extreme ecological impacts that result in the loss of biodiversity and ecosystem services by altering native biodiversity, community structure, composition, and functions (Negi et al., 2019; Broda, 2020).

Taking that into consideration, two noxious weed species, *L. camara* L. (Verbenaceae) and *A. conyzoides* L. (Asteraceae), with antifungal properties, were chosen for the study with the goal of bio-prospecting weed extracts for providing an economical and environmentally friendly biopreservation platform in *L. coromandelica* (Houtt.) Merr. against *Laetiporus sulphureus* (Bull.: Fr.) Murr., a common wood-rotting fungus.

## Materials and Methods

### Experimental Location

The experiment was conducted in the Department of Forest Products, Dr. Y. S. Parmar University of Horticulture and Forestry, Nauni, Solan (H.P.) (30.8613° N, 77.1708° E) located at 1,275 m a.s.l.

### Wood Material

The wood blocks [5.0 cm (longitudinal) × 2.5 cm (radial) × 2.5 cm (tangential)] from air-dried sapwood of *L. coromandelica* were prepared at the sawmill workshop of the Department of Forest Products, Nauni, Solan (Himachal Pradesh, India). The blocks were oven-dried at 105 ± 2°C until constant weight is attained before the subsequent treatments. No observable signs of defects, infection by mold, or wood-destroying fungi and termite were detected on wood samples. The density of the oven-dried specimens was in the range of (0.56 to 0.77) (Gupta et al., 2016). To ensure maximum uptake of the treatment solutions, none of the surfaces of the wood samples was sealed. Three replicates, each with six wood blocks, were cut for the tests, along with control samples.

### Plant Material and Extracts Preparation

Two obnoxious weeds, viz., *A. conyzoides* L. and *L. camara* L. were collected from University premises. The botanical identity was confirmed by comparing with the herbarium specimens and a voucher specimen with accession number 7307 and 12674, respectively, deposited at the Herbarium of Department of Forest Products. PE and M were used as extraction solvents for comparison in this study. PE was selected because of its low polarity and ability to remove oils, fats, sterols, and terpenes (Pramod et al., 2017). On the other hand, M is known for its low toxicity, high polarity, and efficiency in extracting various polar phytocompounds (phenolics, flavonoids, and so on) (Swamy et al., 2015). The grounded leaves of *L. camara* and aerial parts of *A. conyzoides* (100.0 g each) were extracted sequentially first with refluxing PE (Sigma-Aldrich, St. Louis, MO) in a Soxhlet extractor for 10 cycles. The residual PE was allowed to evaporate from the material and was subsequently extracted with refluxing M (Merck, India). The solvents were separated from the respective solution by rotary evaporation and preserved separately at 4°C until further use. The extracts were named E1 (*L. camara* PE extract), E2 (*L. camara* M extract), E3 (*A. conyzoides* PE extract), E4 (*A. conyzoides* M extract). The soxhlet extractions were replicated thrice.

### Treatment Method

The surface-applied treatment steeping was conducted. The PE and M extracts of *Lantana* (L) and *Ageratum* (A) were prepared at different concentrations of 0.25, 0.50, 1.00, 1.50, and 2.00% (w/v) by diluting the respective amount of extract in 5% M (v/v). The oven-dried wood blocks (M_1_) (six in each replicate) were immersed in a solution of each concentration of extracts for 72 h at room temperature. After the treatment, the excess extract was wiped off the surface of the wood blocks, and the wet weight (M_2_) and dimensions of all the treated wood blocks were evaluated. The retentions for each treatment were calculated according to Equation (1):

(1) $$\begin{array}{r} R=\frac{G\;\times C}{V}\;\times 10Kgm^{-3} \end{array}$$

where G (M_2_-M_1_) is the grams of treatment solution absorbed by the wood block; C is the Grams of preservative solutions in 100 g of the treatment solution; V is the volume of wood block in cubic centimeters.

All the wood blocks were oven-dried at (103- ± 2°C; 24 h). The treated and untreated samples were conditioned at 20 ± 2°C and 65 ± 5% relative humidity (RH). The dimensions and weight (M_3_) were recorded and evaluated for weight percent gain (WPG) (Equation 2):

(2) $$\begin{array}{r} WPG\;\left(\%\right)=\frac{M_{3}-M_{1}}{M_{1}}\times 100 \end{array}$$

where M_3_ is the oven-dried weight of the sample after treatment; M_1_ is the oven-dried weight of the sample before treatment.

### Dimensional Stability

The dimensional stabilities of the treated and untreated wood samples were determined by measuring swelling (S_w_) and shrinkage (S_h_) [tangential (T), radial (R) direction], T/R shrinkage ratio, volumetric swelling (VS), volumetric swelling (S), and anti-shrink efficiency (ASE). The oven-dried treated and untreated wood blocks (conditioned before) were soaked for 1 week in distilled water at room temperature until completely saturated. Then, the wood blocks were removed, and the dimensions in wet condition were recorded. The treated and untreated samples were conditioned at 20 ± 2°C and 65 ± 5% RH followed by oven-dried at 103 ± 2°C to a constant weight, and the dimensions were recorded again to the nearest millimeter with a digital Vernier caliper. Percentage swelling and shrinkage in tangential and radial directions were measured using (Equation 3) and (Equation 4):

(3) $$\begin{array}{r} S_{w}\left(\;\%\;\right)=\frac{\left(D_{s}-D_{o}\right)}{D_{o}}\times 100 \end{array}$$

(4) $$\begin{array}{r} S_{h}\left(\;\%\;\right)=\frac{\left(D_{s}-D_{oo}\right)}{D_{s}}\times 100 \end{array}$$

where D_s_ is dimension at saturation; D_o_ is dimension at oven-dried condition before saturation; D_oo_ is dimension at oven-dried condition after saturation.

The T/R shrinkage ratio, according to Priadi et al. (2020) (Equation 5),

(5) $$\begin{array}{r} T/R=\frac{Tangential\;shrinkage\;\left(\%\right)}{Radial\;shrinkage\;\left(\%\right)}\;\times 100 \end{array}$$

The volumetric swelling coefficient (S) (Equation 6), volumetric shrinkage coefficient (VS) (Equation 7), and anti-shrink efficiency (ASE) (Equation 8) were calculated according to Rowell and Ellis (1978):

(6) $$\begin{array}{r} S\;=\frac{V_{2}-V_{1}}{V_{1}}\;\times 100 \end{array}$$

where V_2_ = volume of wood sample after at wet condition (cm^3^); V_1_ = volume of oven-dried samples before wetting (cm^3^).

(7) $$\begin{array}{r} VS\;=\frac{V_{2}-V_{1}}{V_{2}}\;\times 100 \end{array}$$

where V_2_ = volume of wood sample after at wet condition (cm^3^); V_1_ = volume of oven-dried samples after wetting (cm^3^).

(8) $$\begin{array}{r} ASE\;=\frac{S_{1}-S_{2}}{S_{1}}\;\times 100 \end{array}$$

where S_1_ is the volumetric shrinkage coefficient of untreated wood samples and S_2_ is the volumetric shrinkage coefficient of treated wood samples.

### Fungal Strain

*Laetiporus sulphureus* (MTCC 1067), used in the study, was procured from the Microbial Type Culture Collection and Gene Bank (MTCC), CSIR-Institute of Microbial Technology, Chandigarh, India. Fresh colonies of the fungi were grown in 4% malt powder and 2% agar culture media in a growing chamber at 20 ± 2°C and 65 ± 5% relative humidity. Once the mycelia covered the whole Petri dish, the colonies were refrigerated (8°C), and 2 days before their further use, the colonies were returned to the growing chamber at 20 ± 2°C and 65 ± 5% relative humidity.

### Antifungal Assay

The method of Falck (1907) and Xie et al. (2017) with slight modifications was employed for antifungal evaluation of PE and M extracts of *L. camara* and *A. conyzoides*, which were tested at 0.25, 0.50, 1.00, 1.50, and 2.00% concentrations against *L. sulphureus* in 9 cm Petri dishes. Double strength potato dextrose agar (PDA) medium was prepared by doubling the amount of constituents except distilled water, and the medium was sterilized at 1.05 kg/cm^2^ pressure for 20 min. Simultaneously, double concentrations of different plant extracts were also prepared in sterilized distilled water to get desired concentration of plant extracts after mixing the fungicide solutions in the double strength media. Plant extract solutions were added separately to equal quantities of double-strength PDA medium aseptically before pouring in Petri plates. These plates were then inoculated with the 7-day-old culture of the fungus. A control treatment was also maintained in which only plain sterilized water was added to the double strength medium. Each treatment was replicated thrice, and the inoculated plates were incubated at 27 ± 1°C in a BOD incubator for 5–7 days. The colony diameter of test fungi was recorded till the mycelia reached the edges of the control plates. The antifungal index was calculated according to Equation (9):

(9) $$\begin{array}{r} Antifungal\;index=\left(1-Da/Db\right)\;\times 100 \end{array}$$

where Da = the diameter of the growth zone in the experimental plate (mm), Db = the diameter of the growth zone in the control plate (mm).

### Decay Test

The block decay test was performed following a modified version of the Sarker et al. (2006). The glass jars (500 mL) containing growth media (100 mL), 2% malt powder, and 2% agar were inoculated with one plug (Ø 5.5 mm) of the actively growing *L. sulphureous* under sterile conditions. The jars were sealed with parafilm and incubated at 25 ± 1°C and 65 ± 5% RH till the colonies reached the edge of the glass jars. The treated as well as untreated wood blocks were exposed to fungus by placing them on mycelia grown in the jars and incubated again at 25 ± 1°C and 65 ± 5% RH for 21 days for visual observation of the fungal growth by the naked eye using the following scale:

**Table:** 

**Disease Rating Scale**	**Surface coverage**
0	0%
1	1–25%
2	26–50%
3	51–75%
4	>76%

Percent fungus colonization index and growth inhibition were calculated according to McKinney (1923) (Equation 10) and Vincent (1947) (Equation 11), respectively.

(10) $$\begin{array}{r} Percent\;fungus\;colonization\;Index=\\ \;\frac{Sum\;of\;all\;disease\;ratings}{Total\;number\;of\;ratings\;\times Maximum\;grade}\;\times 100\; \end{array}$$

(11) $$\begin{array}{r} Fungus\;growth\;inhibition\;\left(\%\right)=\frac{C-T}{C}\times 100 \end{array}$$

where C = fungus colonization in control (%) and T = fungus colonization in treated wood (%).

These samples were continued to be incubated at 25 ± 1°C and 65 ± 5% RH for 12 weeks. After exposure, the samples were then taken out of the jars, and hyphae from the surface of the wood were gently removed with a brush. Wood samples were oven-dried at 103 ± 3°C for 24 h and weighed to calculate the mass loss according to Equation (12):

(12) $$\begin{array}{r} ML\;\left(\%\right)=\frac{M_{0}-M_{1}}{M_{0}}\times 100 \end{array}$$

where ML is the mass loss (%), M_0_ is the oven-dry weight of the sample before fungi test (g), and M_1_ is the oven-dried weight after fungi test (g).

### Statistical Analysis

The data recorded from the laboratory experiments were subjected to the statistical ANOVA using SPSS Version 25.0 (IBM SPSS Statistics for Windows, IBM Corporation, Armonk, NY) following Completely randomized block design (factorial). Means were expressed as mean ± SE and grouped using Tukey's honestly significant difference test at a significance level of 0.05.

## Results

### Extractive Retention and WPG

The data pertaining to the retention of *L. camara* and *A. conyzoides* extracts at different concentrations in *L. coromandelica* wood blocks are presented in Table 1. With increasing concentration, the extract retentions were found to increase significantly (*p* < 0.01). The wood blocks treated with the highest concentration (2.00%) of plant extracts retained 7.45 kg/m^3^ (E1), 7.37 kg/m^3^ (E2), 6.75 kg/m^3^ (E4), and 6.08 kg/m^3^ (E3). Whereas, lowest retentions (0.76 kg/m^3^, E4; 0.95 kg/m^3^, E1; 1.08 kg/m^3^, E2; 1.12 kg/m^3^, E3) was observed at 0.25% concentration of plant extracts. Whereas, lowest retentions (0.76 kg/m^3^, E4; 0.95 kg/m^3^, E1; 1.08 kg/m^3^, E2; 1.12 kg/m^3^, E3) was observed at 0.25% concentration of plant extracts. The data on wood retention were in line with that of WPG. In general, with increasing extract concentration an increase in WPG was observed (*p* < 0.01) (Table 1). Similarly, as reported for retention data, negative WPG were observed at low concentrations of 0.25 and 0.50%, while highest at 2.00% concentration.

**Table 1:** Retention (kg/m^3^), weight percent gain (WPG, %), and mass loss (ML, %) of untreated and plant extracts treated *Lannea coromandelica* wood blocks.

**Treatment**	**Concentration (%)**	**Retention (Kg/m^**3**^)**	**WPG (%)**	**Mass Loss (ML) (%)**
Untreated/Control	/	/	/	31.60 ± 0.28^k^
E1	0.25	1.12 ± 0.01^a^	−2.46 ± 0.22^ab^	28.24 ± 0.09^hi^
	0.50	2.17 ± 0.10^b^	−0.88 ± 0.02^c^	27.06 ± 0.23^fgh^
	1.00	4.31 ± 0.02^c^	0.68 ± 0.21^de^	25.71 ± 0.05^ef^
	1.50	7.36 ± 0.06^d^	1.17 ± 0.11^e^	19.40 ± 0.05^b^
	2.00	7.45 ± 0.12^d^	1.39 ± 0.09^e^	16.43 ± 0.15^a^
E2	0.25	1.08 ± 0.01^a^	−2.13 ± 0.11^ab^	29.11 ± 0.31^ij^
	0.50	2.20 ± 0.10^b^	−0.97 ± 0.25^c^	28.99 ± 0.35^ij^
	1.00	3.54 ± 0.01^c^	1.08 ± 0.13^e^	26.50 ± 0.45^fg^
	1.50	5.19 ± 0.07^d^	1.15 ± 0.09^e^	24.78 ± 0.43^de^
	2.00	7.37 ± 0.09^e^	1.18 ± 0.06^e^	21.78 ± 0.35^c^
E3	0.25	0.95 ± 0.01^a^	−1.89 ± 0.11^b^	27.73 ± 0.42^ghi^
	0.50	1.94 ± 0.11^b^	−0.79 ± 0.19^c^	26.77 ± 0.34^fgh^
	1.00	3.84 ± 0.04^c^	0.16 ± 0.10^d^	24.96 ± 0.14^e^
	1.50	6.07 ± 0.12^d^	0.68 ± 0.05^de^	19.61 ± 0.21^b^
	2.00	6.09 ± 0.15^d^	0.74 ± 0.04^de^	17.11 ± 0.25^a^
E4	0.25	0.76 ± 0.02^a^	−2.77 ± 0.14^a^	29.90 ± 0.38^j^
	0.50	1.51 ± 0.03^b^	−0.59 ± 0.04^c^	27.89 ± 0.35^ghi^
	1.00	3.56 ± 0.06^c^	0.80 ± 0.16^de^	24.80 ± 0.32^de^
	1.50	4.74 ± 0.15^d^	0.81 ± 0.07^e^	22.96 ± 0.06^d^
	2.00	6.75 ± 0.22^e^	0.96 ± 0.03^de^	21.68 ± 0.06^c^

*Data represent the mean of three replicates ± Standard error (SE). Different superscripts letters in a column specify a statistically significant difference between the means (P ≤ 0.05, Turkey's HSD Test)*.

### Dimensional Stability

**Figure 1:**
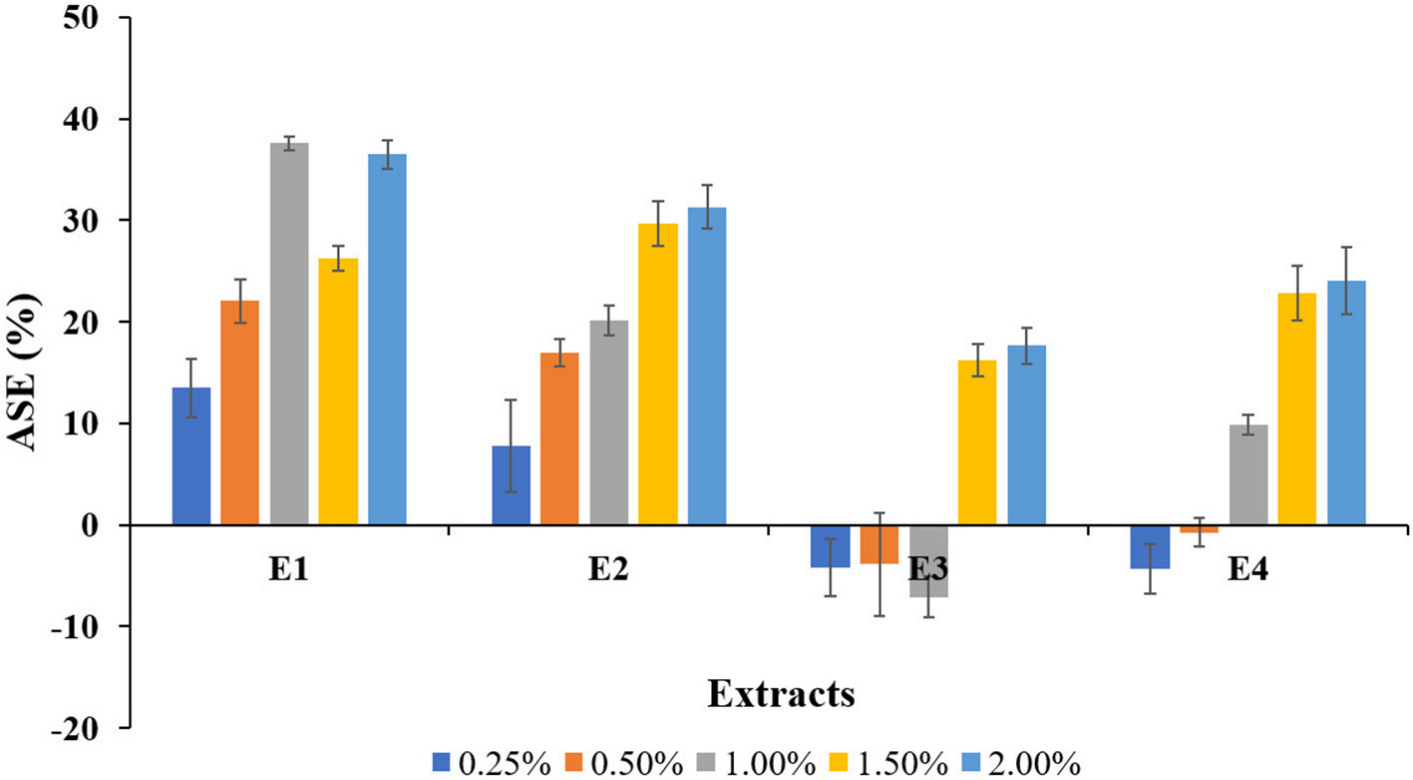
Anti-shrink efficiency (ASE) values for the plant extracts treated wood blocks of *Lannea coromandelica*.

The relative dimensional changes [swelling (Sw) and shrinkage (Sh)] in untreated and treated wood blocks of *L. coromandelica* are presented in Table 2. The results revealed significant differences for swelling and shrinkage in radial and tangential directions and volumetric coefficient (*p* < 0.01). The greater dimensional changes of untreated wood in the tangential direction (Sw: 4.24%; Sh: 3.62%) than in the radial direction (Sw: 3.98%; Sh: 3.30%) were observed (due to the small dimensional changes of the examined samples, being in the range 0.2–0.4%, measurements on swelling and shrinkage in the longitudinal direction were not given). The treatments with E1 and E2 significantly reduced the moisture-dependent dimensional changes in all directions over control. The lowest radial (2.75%; 1.50%), tangential (2.70%; 1.00%), and volumetric swelling (6.44%; 0.25%) values were obtained for E2. The highest radial (6.00%; 1.50%), tangential (6.30%; 2.00%), and volumetric swelling (13.17%; 1.50%) values were measured for E3. The swelling of wood blocks in radial and tangential directions increased with an increase in extract concentration while the wood shrinkage followed a reducing trend. The minimum shrinkage values for radial (2.01%), tangential (2.09%) direction, and volumetric swelling (4.57%) values were obtained for E1 at 2.00% concentration. The maximum radial (3.73%; 0.25%), and volumetric shrinkage (7.71%; 1.00%) values were recorded for E3, whereas the highest tangential shrinkage (4.10%; 1.50%) for E4. The percentage of shrinkage reduced (ASE) as a result of extracts treatment (*p* < 0.05) is presented in Figure 1. In comparison with the control, there was more variability between different plant extracts and among blocks treated with the same plant extract. The ASE for E1 and E2 treatments were registered with positive values, and the maximum ASE (37.57%) was registered for E1 at 1.00% concentration and lowest (7.77%) for E2 at 0.25%. The ASE values obtained for E3 and E4 treatments were generally lower than those obtained for previous treatments, with negative values ranging from −7 to −1% obtained at low concentrations. The other positive results for E3 and E4 ranged between a minimum of 9.88% (1.00%; E4) and a maximum of 24.06% (2.00%; E4).

**Table 2:** Dimensional stability (swelling and shrinkage) of untreated and plant extracts treated wood blocks of *Lannea coromandelica*.

**Treatment**		**Swelling**			**Shrinkage**		
	**Concentration (%)**	**Swelling (R, %)**	**Swelling (T, %)**	**Volumetric swelling (%)**	**Shrinkage (R, %)**	**Shrinkage (T, %)**	**Volumetric Shrinkage (%)**
Untreated/Control	/	3.98 ± 0.11^abcd^	4.24 ± 0.04^bcde^	9.09 ± 0.15^cde^	3.30 ± 0.08^bcde^	3.62 ± 0.03^bcde^	7.20 ± 0.08^ghi^
E1	0.25	2.88 ± 0.07^abc^	3.03 ± 0.36^abc^	6.64 ± 0.73^ab^	3.24 ± 0.22^abcde^	2.89 ± 0.28^abcde^	6.22 ± 0.16^def^
	0.50	3.03 ± 0.36^abcd^	3.18 ± 0.14^abc^	6.66 ± 0.24^ab^	2.47 ± 0.06^abcd^	2.81 ± 0.11^abcde^	5.62 ± 0.20^bcde^
	1.00	3.34 ± 0.17^ab^	3.37 ± 0.17^abc^	7.27 ± 0.11^abc^	2.47 ± 0.09^abcd^	2.72 ± 0.08^abcde^	4.49 ± 0.04^a^
	1.50	3.57 ± 0.10^abcd^	3.71 ± 0.20^abcde^	7.92 ± 0.31^abcd^	2.28 ± 0.16^abc^	2.37 ± 0.28^abc^	5.31 ± 0.12^abcd^
	2.00	4.09 ± 0.33^abcd^	3.98 ± 0.32^abcde^	8.74 ± 0.09^bcde^	2.01 ± 0.16^a^	2.09 ± 0.34^a^	4.57 ± 0.06^a^
E2	0.25	3.63 ± 0.13^abcd^	2.88 ± 0.12^ab^	6.72 ± 0.03^a^	3.05 ± 0.07^abcde^	3.24 ± 0.15^abcde^	6.63 ± 0.25^fgh^
	0.50	3.21 ± 0.38^abcd^	3.40 ± 0.10^abcd^	6.88 ± 0.34^ab^	3.03 ± 0.28^abcde^	2.86 ± 0.09^abcde^	5.98 ± 0.08^cdef^
	1.00	3.05 ± 0.11^abc^	2.70 ± 0.24^a^	7.20 ± 0.15^abc^	2.53 ± 0.11^abcde^	2.65 ± 0.21^abcde^	5.75 ± 0.06^bcdef^
	1.50	2.75 ± 0.07^a^	3.45 ± 0.12^abcd^	7.31 ± 0.28^abc^	2.55 ± 0.12^abcde^	2.64 ± 0.03^abcde^	5.06 ± 0.13^abc^
	2.00	3.90 ± 0.14^abcd^	4.43 ± 0.15^cde^	9.09 ± 0.03^cde^	2.17 ± 0.11^ab^	2.20 ± 0.21^ab^	4.95 ± 0.21^ab^
E3	0.25	4.36 ± 0.27^cd^	4.80 ± 0.44^de^	10.02 ± 0.72^def^	3.73 ± 0.22^e^	3.87 ± 0.04^de^	7.50 ± 0.21^hi^
	0.50	4.49 ± 0.18^d^	4.91 ± 0.26^e^	10.23 ± 0.57^ef^	3.61 ± 0.13^de^	3.08 ± 0.47^abcde^	7.47 ± 0.28^hi^
	1.00	4.51 ± 0.31^d^	5.10 ± 0.31^ef^	11.65 ± 0.30^fg^	2.74 ± 0.34^abcde^	2.94 ± 0.21^abcde^	7.71 ± 0.08^i^
	1.50	6.00 ± 0.22^e^	6.25 ± 0.36^f^	13.17 ± 0.59^g^	2.61 ± 0.43^abcde^	2.57 ± 0.47^abcd^	6.03 ± 0.04^def^
	2.00	5.98 ± 0.26^e^	6.30 ± 0.26^f^	13.13 ± 0.27^g^	2.50 ± 0.09^abcde^	2.43 ± 0.03^abcde^	5.93 ± 0.18^cdef^
E4	0.25	4.10 ± 0.06^abcd^	4.21 ± 0.17^bcde^	9.26 ± 0.13^cde^	3.47 ± 0.09^cde^	3.75 ± 0.25^cde^	7.51 ± 0.12^hi^
	0.50	4.18 ± 0.11^bcd^	4.36 ± 0.04^cde^	9.27 ± 0.08^cde^	3.23 ± 0.30^abcde^	3.72 ± 0.08^cde^	7.26 ± 0.15^ghi^
	1.00	4.35 ± 0.25^cd^	4.40 ± 0.02^cde^	9.38 ± 0.05^cde^	3.26 ± 0.14^bcde^	3.77 ± 0.13^cde^	6.49 ± 0.03^efg^
	1.50	4.32 ± 0.28^cd^	4.41 ± 0.02^cde^	9.50 ± 0.29^de^	2.28 ± 0.07^abc^	4.10 ± 0.15^e^	5.55 ± 0.15^bcd^
	2.00	4.39 ± 0.06^cd^	5.01 ± 0.21^e^	10.17 ± 0.47^ef^	2.08 ± 0.19^ab^	4.06 ± 0.30^e^	5.47 ± 0.08^bcde^

*Data represent the mean of three replicates ± SE. Different superscripts letters in a column specify a statistically significant difference between the means (P ≤ 0.05, Tukey's HSD test)*.

### *In vitro* Antifungal Assay

**Figure 2:**
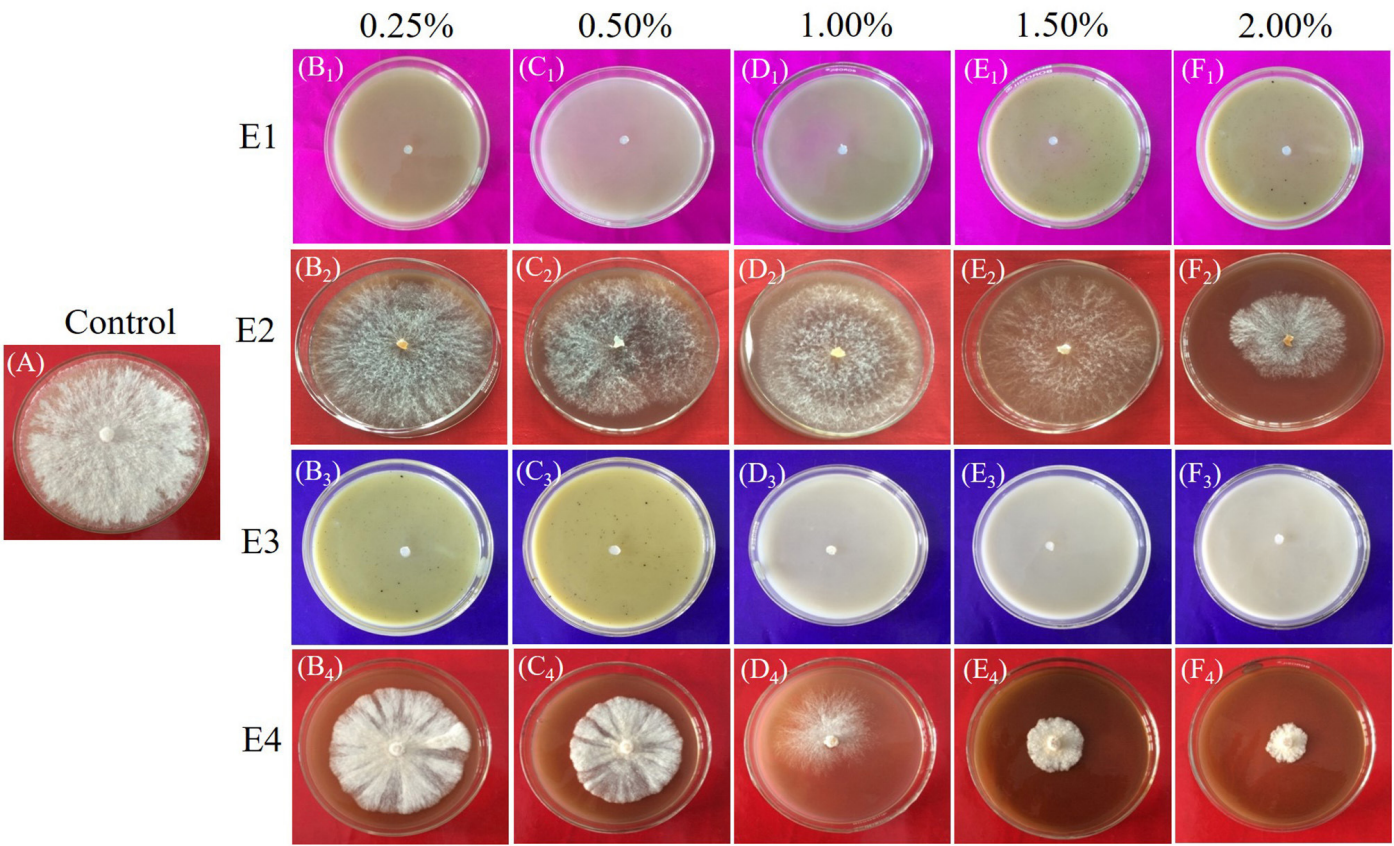
**(A–F)** Antifungal activity of *Laetiporus sulphureus* against **(A)** Control (PDA+ test fungi) and at 0.25, 0.50, 1.00, 1.50, and 2.00% concentrations of extracts (PDA+ extracts+ test fungi) E1 **(B**_**1**_**-F**_**1**_**)**; E2 **(B**_**2**_**-F**_**2**_**)**; E3 **(B**_**3**_**-F**_**3**_**)**; and E4 **(B**_**4**_**-F**_**4**_**)**.

**Figure 3:**
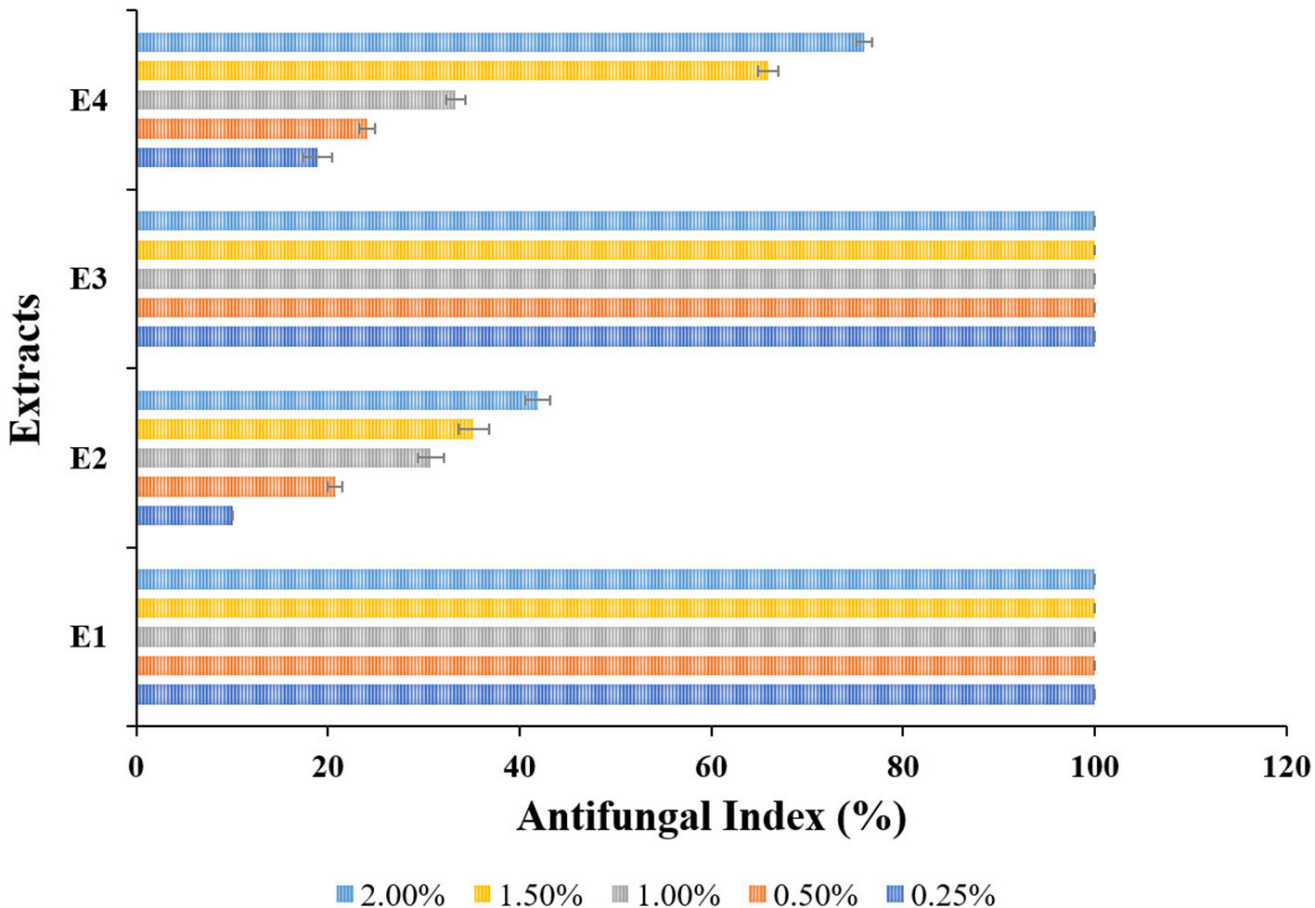
Antifungal index (%) of different extracts from *Lantana camara* (E1, E2) and *Ageratum conyzoides* (E3, E4) against *Laetiporus sulphureus* at 0.25, 0.50, 1.00, 1.50, and 2.00% concentrations.

The antifungal activity of the selected plant of *L. camara* (E1, E2) and *A. conyzoides* (E3, E4) extracts against the wood-rotting fungus *L. sulphureus* were shown in Figure 2 (antifungal assay) and Figure 3 (antifungal index). The results revealed that E1 (Figure 2) as well as E3 (Figure 2) showed the best activity against *L. sulphureus* and induced 100% antifungal index at all the test concentrations. In contrast, the other extracts, i.e., E2 (Figure 2) and E4 (Figure 2), showed relatively lower activity. E4 exhibited the highest antifungal index of 75.93% at 2.00% concentration, but E2 (Figures 2, 3) expressed reduced antifungal activities at the same concentration with the antifungal index lower than 41.85%. Despite greater variation between concentrations, the extracts (E2; Figure 2, E4; Figure 2) demonstrated dose-dependent antifungal activity that increased with increasing concentrations from 0.25 to 2.00%. The least antifungal activities of extracts E2 (Figure 2) and E4 (Figure 2) were observed at the lowest concentration (0.25%) with an antifungal index lower than 20% (10.04 and 18.89%, respectively).

### Visual Observations

**Figure 4:**
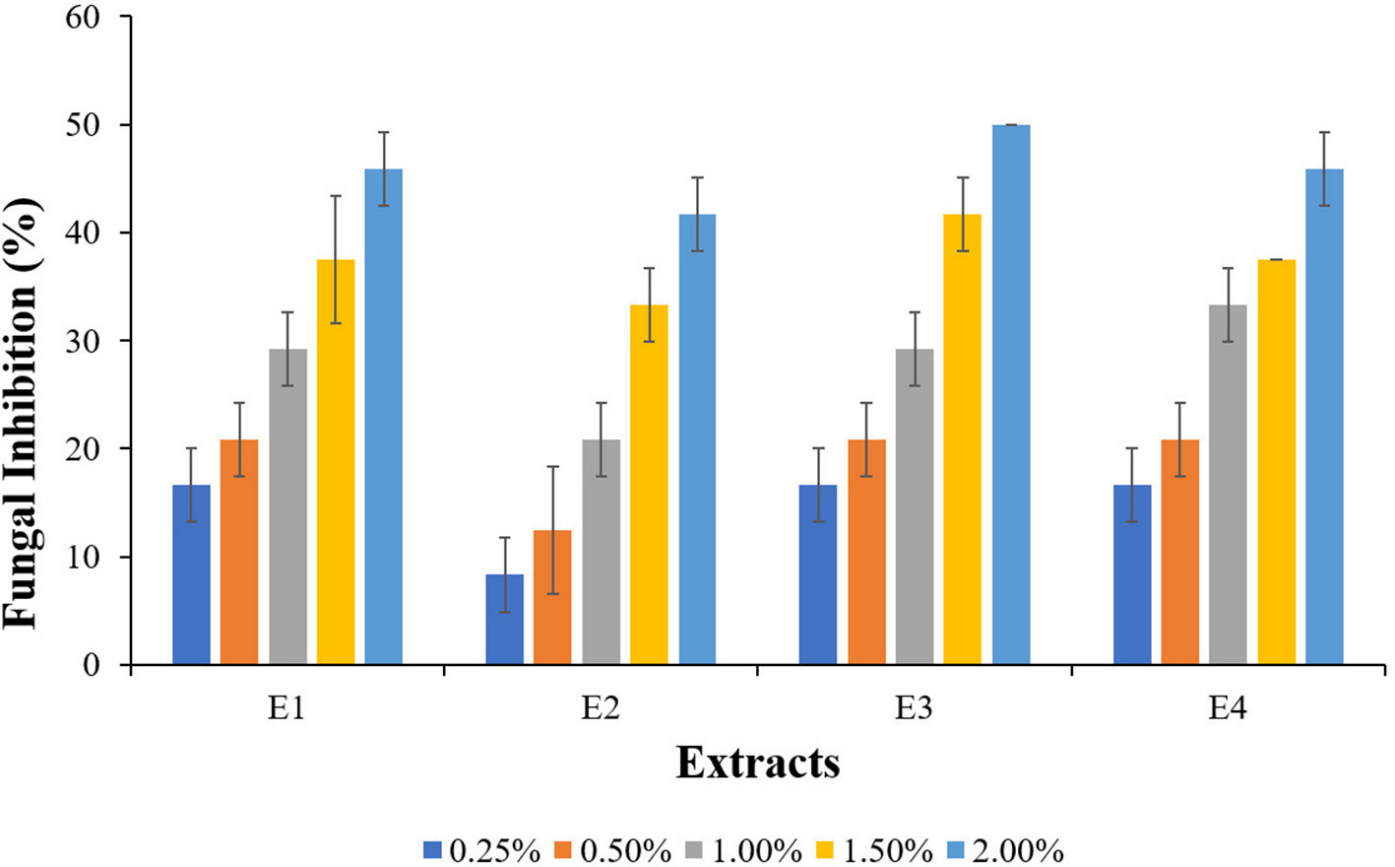
Fungal inhibition in plant extracts treated wood blocks of *Lannea coromandelica*.

Visual observation of mycelium growth on the wood surface was used to assess the antifungal activities of *L. camara*, and *A. conyzoides* extracts treated wood blocks. The results of fungal inhibition determined after 21 days of incubation are shown in Figure 4. L. sulphureous grew quickly on untreated wood blocks, and the surface of the wood blocks was completely overgrown (rating scale 5), indicating complete fungal colonization (100%). However, the types and concentrations of extracts had a significant effect on the mycelial linear growth of test fungi. They showed remarkable inhibition effects over the control. Furthermore, an increase in fungal inhibition was observed with increasing extract concentrations. The PE extracts of both plants (E1 and E3) inhibited the growth of the test fungi significantly, with the highest fungal inhibition (50.00%) recorded for E3 at 2.00% concentration and the lowest (16.77%) recorded for E1 and E3 at 0.25% concentration. While the effect of methanolic extracts (E2 and E4) was lower in comparison, test fungi inhibition was found to be 46% (E2; 8.33% and E4; 45.00%).

### Resistance Against Decay Fungi

The mean percentage mass loss (ML) of extract-treated *L. coromandelica* wood blocks tested after exposure to *L. sulphureous* for 12 weeks (*p* < 0.01) are shown in Table 1. The highest ML (31.60%) was recorded in untreated wood blocks. Both the plants improved the wood resistance as the treated wood blocks showed an ML of <30%. The blocks treated with PE extracts (E1 and E3) displayed more decay resistance with ML as ranged between (16–28%) compared with 21–30% for M extracts (E2 and E4). The fungal resistance improved with increasing concentration from 0.25 to 2.00%. The minimum ML (16.77%) was recorded in E1 at 2.00% concentration, representing >45% reduction in ML over control followed by E3 (17.11%). Although the M extracts (E2 and E4) were comparatively less effective, but offered decay resistance higher than the control with the lowest ML (21.68%) at 2.00% concentration.

## Discussion

### Extractive Retention and WPG

The retention of the preservative is a significant factor demonstrating the quality of impregnation (Dong et al., 2020). Retention is primarily determined by treatment conditions (such as duration and preservative type) and concentration. The variation in retention was observed with extract type, with E1 having the best retention. E1 appears to have sufficient viscosity to allow for good penetration into the wood. With increasing concentrations of plant extracts, the retention of wood blocks has increased. Several authors have discussed the linear relationship between increasing preservative retention and increasing preservative concentration (Syazwan et al., 2017; Ouyang et al., 2018; Shukla et al., 2019). The heterogeneous texture, density, and porosity of wood species significantly impact retention values, whereas the particle size of the extracts is responsible for the penetration efficiency of the retention rate. The extract retention data agreed with WPG regarding the amount of extractive that entered the wood blocks (lumen and/or cell wall). The variations in WPG can be attributed to moisture content, variation in wood density, and absorption and intensity of interaction of extracts with different wood elements present in the microstructure of *L. coromandelica* wood. On the other hand, negative retention can be related to extractives leached from wood during treatment with extract solutions. While investigating the potential use of the Maillard reaction to modify wood, Peeters et al. (2018) reported similar incompetent treatments with MgCl_2_, in that the weight percentage gains were negative (as much as 5 to 6% WPG in the case of glucosamine with magnesium chloride), indicating removal of cell wall components as a result of the reaction.

### Dimensional Stability

Wood swelling is related to increased moisture content, while shrinkage is related to decreased moisture content. Because wood is anisotropic, swelling and shrinkage occur at different rates and magnitudes in different directions, most notably in the tangential direction, followed by the radial direction, and are negligible in the longitudinal direction (Cai et al., 2019). This is because microfibrils in the tangential plane are parallel to the axis of the cell wall, whereas the radial plane has a restraining effect due to the presence of wood rays (radial microfibril orientation) (Elaieb et al., 2019). When compared with untreated wood blocks, the extracts of *L. camara* (E1 and E2) demonstrated reduced dimensional changes (swelling and shrinkage; different planes, volumetric), revealing the ability of these extracts to improve the dimensional stability of *L. coromandelica* wood blocks. The findings are consistent with those of Var and Kardas (2019), that discovered that salt natural geothermal water (SNGW) treatments reduced the swelling values of pine woods. However, the performance of A. conyzoides (E3 and E4) extracts was lower in providing dimensional stability over control. Temiz et al. (2013) reported higher swelling (tangential plane) of *Arundo donax* bio-oil treated samples than control. The increasing tangential and radial swelling with increased extract concentrations are in line with the finding of Shuib (2011), Okon (2014), Bossu et al. (2016), and Ney et al. (2019).

**Figure 5:**
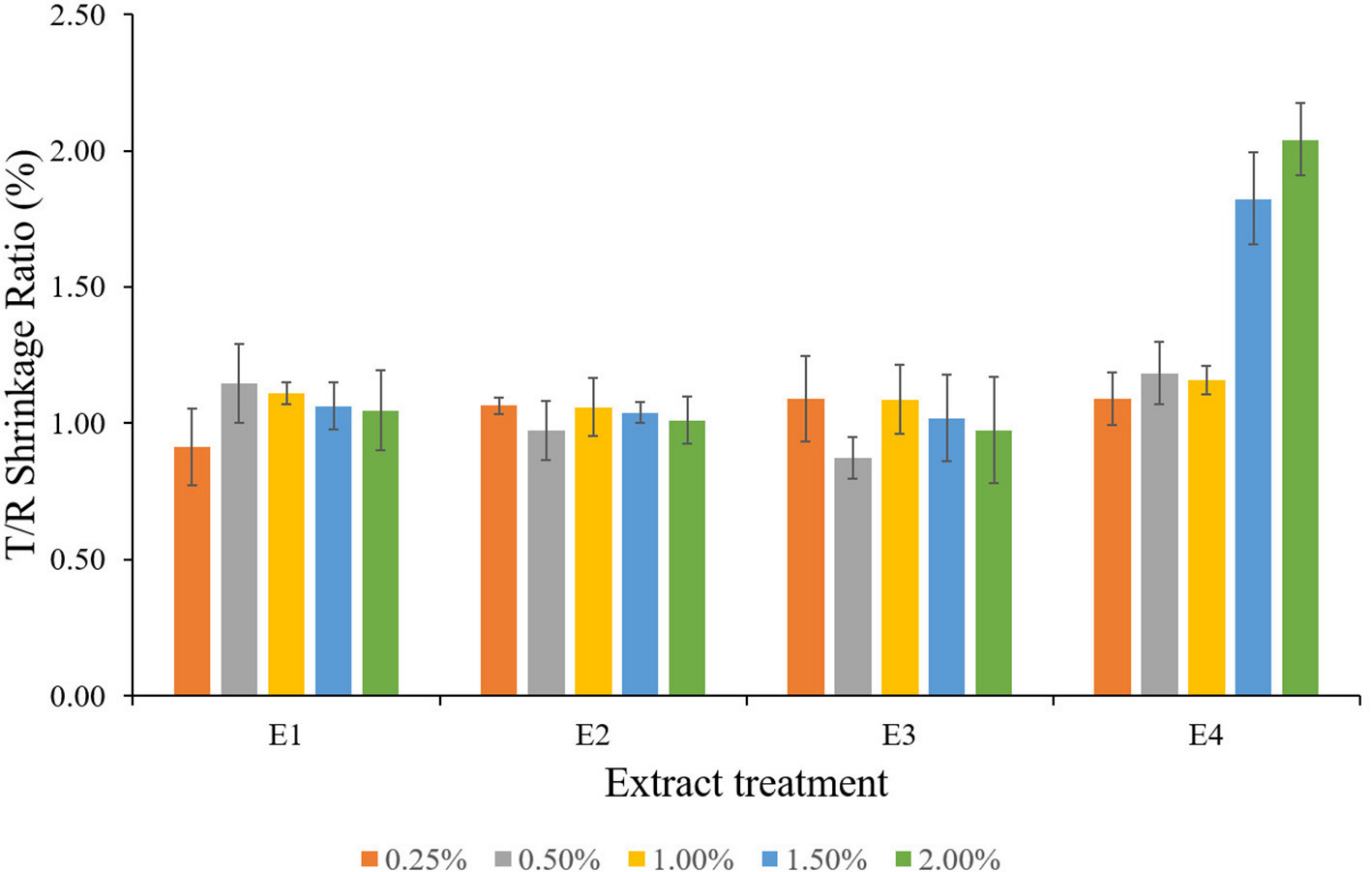
Tangential-radial shrinkage ratio (T/R) of plant extract treated wood blocks of *Lannea coromandelica*.

The extracts of *L. camara* (E1 and E2) reduced the shrinkage values in tangential as well as radial direction with an increase in extract concentration representing reduction improvement of 20–42%; E1, 10–39%; E2 in the tangential direction, and 2–39%; E1, 7–34%; E2 in the radial direction. The shrinkage reduction in the tangential direction was observed to be greater than the reductions in radial directions, which could be due to the vertical orientation of microfibrils in the S2 layer of the cell wall, which is consistent with the findings of Barnett and Bonham (2004) and Okon et al. (2018). The tangential microfibril angle is greater than the radial and longitudinal microfibril angles, resulting in greater tangential shrinkage reduction compared with radial and longitudinal shrinkage (Okon et al., 2018). After treatment, the volumetric swelling of the wood indicated the amount of extract present in the cell wall because the increase in wood volume occurs only after the reagent penetrates the cell wall via pores. Untreated samples had a high volumetric swelling coefficient compared with E1 and E2 extract-treated wood, indicating low cell wall-filling/bulking. Similarly, the plant extract-treated blocks showed a significant increase in volumetric swelling coefficients but decreased volumetric shrinkage coefficients as extract concentration increased. The findings are consistent with those of Salim et al. (2010), Bazyar (2012), and Wu et al. (2012). The tangential-radial shrinkage ratio (T/R ratio) of *L. coromandelica* wood was affected by the extract treatment. The lower T/R ratio of wood treated with E1 and E2 (Figure 5) suggested that *Lantana* extracts (E1 and E2) had a better ability to provide dimensional stability. T/R ratios close to one indicated better dimensional stability of wood, whereas Bowyer et al. (2007) stated that T/R ratios greater than two indicated worse dimensional stability of wood. Priadi et al. (2020) reported similar results while evaluating the dimensional stability, color change, and durability of modified red jabon (*Antochephalus macrophyllus*) wood by double impregnation with boron and methyl methacrylate (MMA) and heat treatment.

ASE compares the volumetric shrinkages of extract-treated blocks to those of untreated blocks to determine the contribution of the extracts to shrinkage suppression. The results clearly showed that treated wood blocks had higher ASE values than untreated ones (ASE = 0), implying that, due to bulking effect, the extracts were effective in reducing shrinkage of wood specimens, thus providing good dimensional stability, as previously explained (Li et al., 2000; Gabrielli and Kamke, 2010). The low and negative values for *A. conyzoides* extracts can be attributed to the fact that the extracts only penetrated a thin layer inside the wood blocks and thus did not counteract shrinkage. The larger dimensional changes will influence the smaller dimensional changes of the untreated samples in the E3, and E4 treated blocks compared with E1 and E2. As a result, rather than concluding a problem with the treated blocks, it can be assumed that the behavior of untreated wood blocks resulted in these lower ASE values. The current findings are consistent with Pepin et al. (2018) and Antonelli et al. (2020).

### Antifungal Activity

As environmental awareness has grown, the focus has shifted to the development of environmentally friendly plant-based wood protectants as an alternative to synthetic wood preservatives. The *in vitro* fungicidal activity of PE (E1 and E3) and M (E2 and E4) extracts against *L. sulphureus* was evaluated to assess the potentials of *L. camara* and *A. conyzoides* for use as wood bioprotectants. All the extracts exhibited remarkable antifungal activity over control, and the PE extracts (E1 and E3) were found to completely inhibit the studied fungi even at the lowest studied concentration. However, M extracts (E2 and E4) were observed to have a comparatively high susceptibility to fungus. The findings are consistent with Linthoingambi and Singh (2013) that observed that the PE leaf extract of *Tithonia diversifolia* had the highest antifungal activity, followed by M and chloroform extracts. Several researchers have reported complete inhibition of *L. sulphureus* (Cheng et al., 2005, 2006; Wang et al., 2005; Xie et al., 2015, 2017). However, the high susceptibility of M extract contradicted the findings of Tripathi et al. (2009); Fayaz et al. (2017). The strong antifungal activities of *L. camara* and *A. conyzoides* plant extracts against a wide variety of fungi have been attributed to the presence of Propanoic acid, 2-hydroxy-, ethyl ester in Cabrido and Demayo (2018); and Precocene II (6,7-dimethoxy-2,2-dimethyl-2-chromene) (Moreira et al., 2007; Adebayo et al., 2010; Yadav et al., 2019) phytochemicals, respectively.

The visual observation of mycelium growth on the wood surface after 21 d of incubation enabled the degree of antifungal activity of the extracts treated wood blocks to be assessed. The findings on fungal inhibition at high extract concentrations of extracts were consistent with the findings of Salem et al. (2016) on fungal inhibition of *P. rigida* heartwood extract at 2% concentration and Mansour and Salem (2015) on fungal inhibition of *Cupressus sempervirens* methanolic extract treated *Acacia saligna* wood *Trichoderma harzianum* at 5%, 10%, and 20% concentrations. In addition to the results obtained, it is also important to highlight that the surface coverage of *L. sulphureous* on the treated wood blocks was less than the controls. When testing wood preservatives, the absence of fungal colonization of the wood was also considered important (Bahmani et al., 2016). Although the extracts demonstrated good antifungal activity after 21 d of incubation, this is only an *in vitro* indicative study, and the experimental conditions did not account for all ecological and endemic factors. Under field conditions, large-scale studies are economically necessary to test the selected plant extracts.

### Decay Test

As a result of biodeterioration of lignocellulosic materials, wood-decaying fungi cause significant losses in wood and other wood-based products (Kositchaiyong et al., 2014; Taghiyari et al., 2014; Xu et al., 2015; Kwaśniewska-Sip et al., 2018). As a result, assessing the resistance of lignocellulosic material to decay fungi and improving the durability of wood and wood-based products is needed to assess the suitability of a timber species for a specific purpose (Sundararaj et al., 2015; Barton-Pudlik et al., 2017). Twelve weeks of exposure to basidiomycetes fungi culminated in the highest ML of untreated *L. coromandelica* control wood blocks, revealing the non-durability of sapwood (Durability class III; field testing, Sundararaj et al., 2015), if not treated with preservatives. The treatment of wood blocks with the selected plant extracts revealed better performance against the test fungi, although the extracts did not provide complete protection. Since the mass loss is relatively less for treated blocks, it can be inferred that the extract might have formed a protective layer over the surface of wood blocks, at least throughout fungal decay (12 weeks). These results were similar to those previously reported by Nayeri et al. (2017), Kwaśniewska-Sip et al. (2018), Ouyang et al. (2018), Izadyar et al. (2020), Ahadnezhad et al. (2021). The differences in ML varied depending on the plant extracts and the concentration used. Despite the fact that all of the wood blocks had complete fungal coverage after 12 weeks of exposure, the resistance provided by PE extracts (E1 and E3) was significantly higher. The results demonstrated a close relationship between ML in the decay test and extracted concentration, with increasing plant extract concentrations reducing decay. The growth of the test fungi on the treated samples was also slowed at low concentrations. Extracts from the selected plant species contained the phytochemicals that slowed fungal attack and reduced weight loss in a susceptible wood species, *L. coromandelica*. The findings of this study are consistent with those stated by Kadir and Hale (2019). The increased resistance of *L. coromandelica* treated wood blocks to test fungi demonstrates the ability of plant extracts to increase durability.

**Figure 6:**
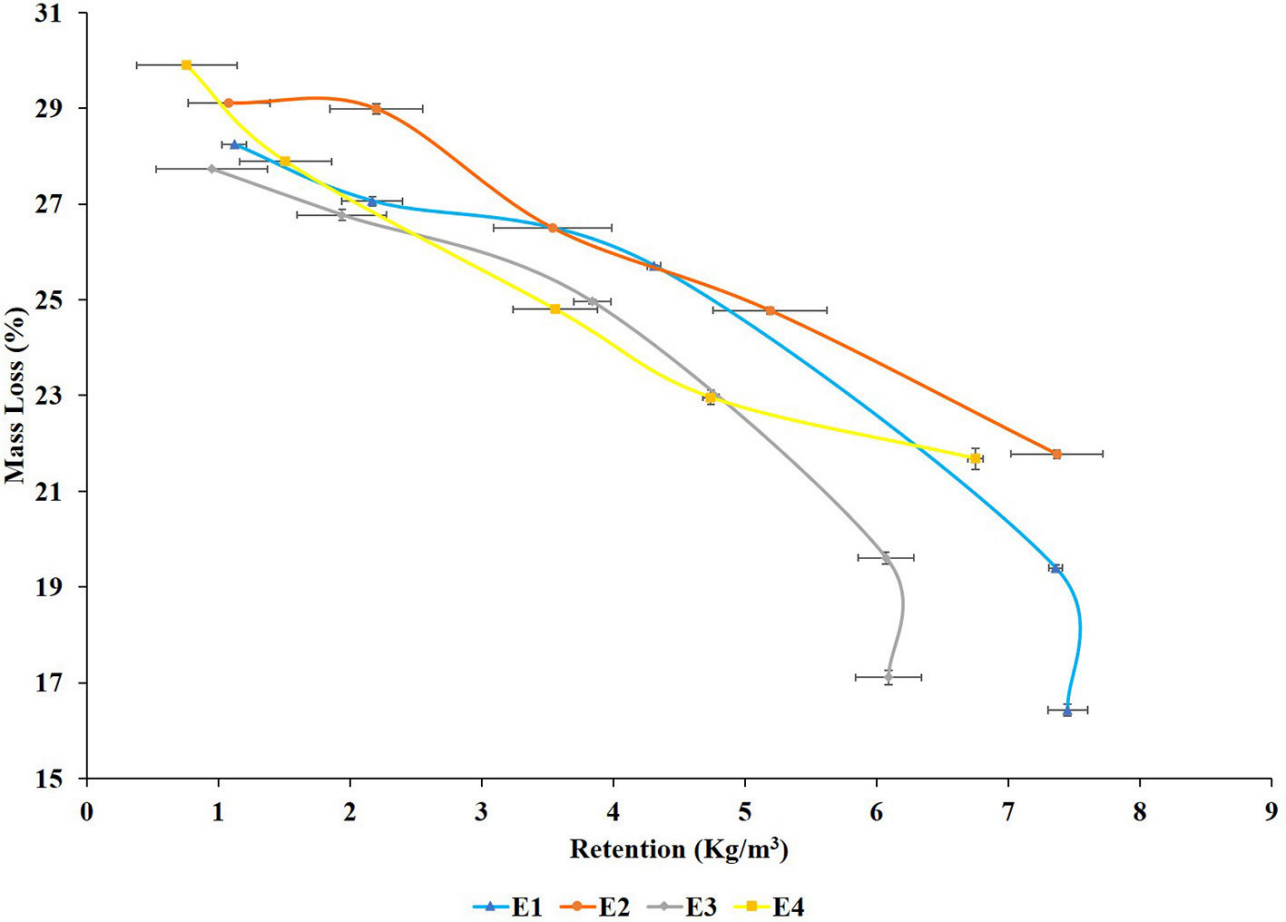
Mass loss concerning extractive retention of *Lannea coromandelica* wood blocks.

**Figure 7:**
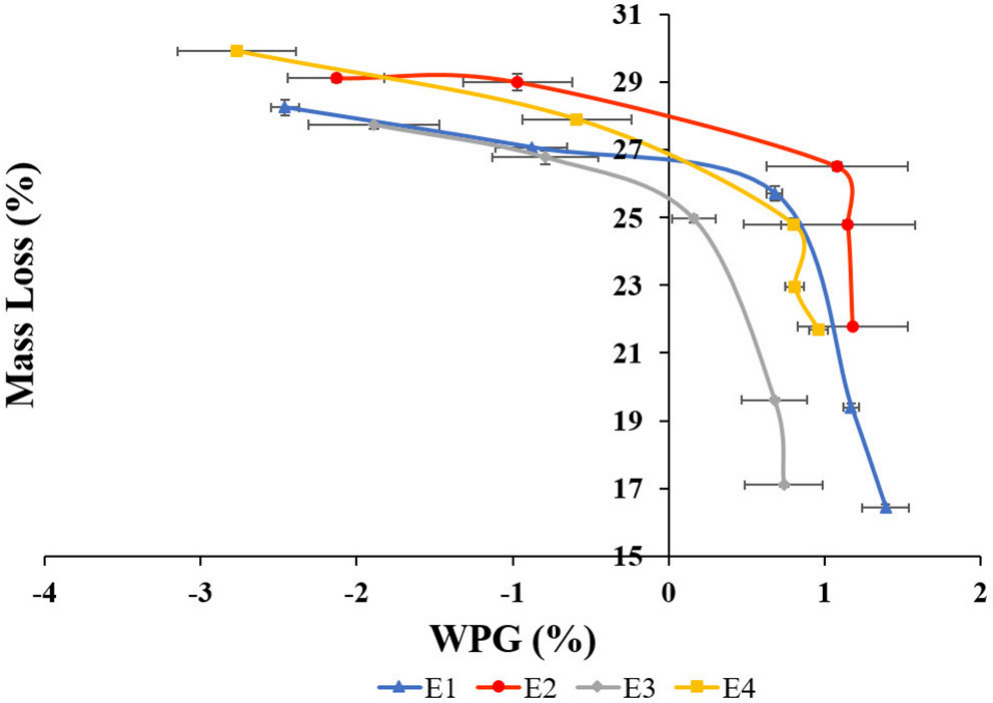
Mass loss concerning weight percent gain (WPG) of *Lannea coromandelica* wood blocks.

**Figure 8:**
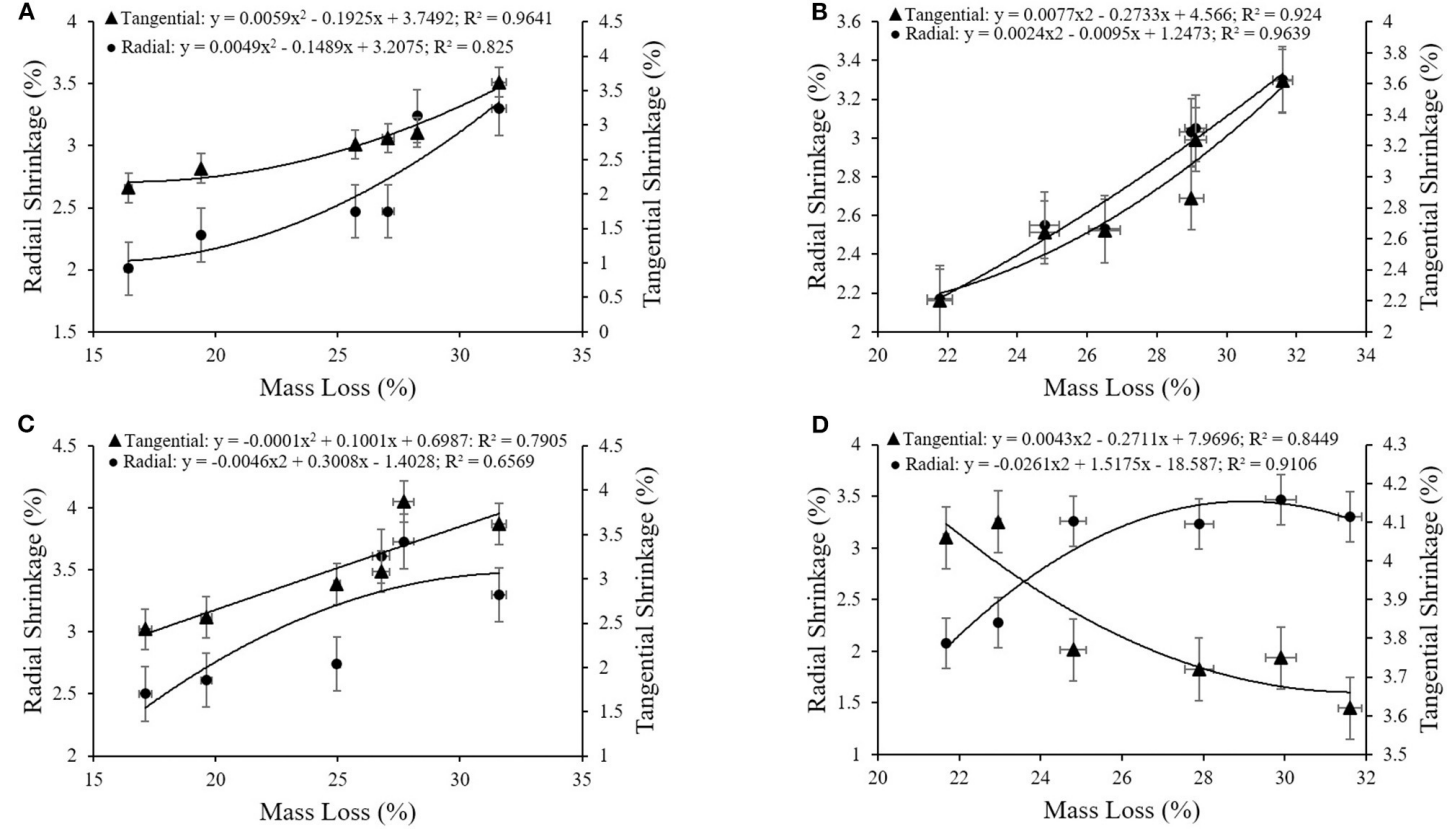
**(A–D)** Relationship between the mass loss and tangential and radial shrinkages for **(A)** E1; **(B)** E2; **(C)** E3; **(D)** E4.

A correlation between retention of extracts (Figure 6), WPG (Figure 7), and ML was also observed as the decay resistance improved with increasing retention and WPG. The results are in line with those reported by Nayeri et al. (2017), Kwaśniewska-Sip et al. (2018), Ouyang et al. (2018), Zelinka et al. (2020). The close relationships between dimensional stability and ML (described by second-degree polynomials) are also confirmed by coefficients of determination. A high correlation was observed between the mass loss and tangential shrinkage for E1 (*R*^2^ = 0.96; Figure 8A) and E2 (*R*^2^ = 0.92; Figure 8B); between mass loss and radial shrinkage for E2 (*R*^2^ = 0.96; Figure 8B) and E4 (*R*^2^ = 0.94; Figure 8D). Whereas, a weaker correlation between mass loss and radial (*R*^2^ = 0.66) and tangential (*R*^2^ = 0.79) shrinkages were recorded for E3 (Figure 8C). A similar high correlation in oak wood buried in waterlogged peat was reported by Babiński et al. (2019).

## Conclusions

Overall, the research findings revealed that treatments with plant extracts increased dimensional stability and decay resistance significantly and also demonstrated their practicable utilization as a preservative. Thus, the extracts have the potential to be developed as a natural fungicide and as a suitable substitute for synthetic preservatives. However, although these plant extracts proved to be effective in laboratory conditions and can be used as an alternative to conventional wood preservatives, more testing is needed to determine their effectiveness in field conditions while considering all ecological and endemic factors. Furthermore, because our study used PE and M extraction, more research is needed to determine the ecotoxicity and efficacy of extractives and their mechanism of inhibition. Furthermore, the technical and economic feasibility of extracting these weed species should be confirmed.

## Data Availability Statement

The original contributions generated for the study are included in the article/supplementary material, further inquiries can be directed to the corresponding author/s.

## Author Contributions

HG and KS conceived the concept. HG, KS, and JS framed the experimental design and wrote and edited the manuscript. HG took the data and conducted statistical analysis. All authors contributed to the article and approved the submitted version.

## Conflict of Interest

The authors declare that the research was conducted in the absence of any commercial or financial relationships that could be construed as a potential conflict of interest.
